# Sensorimotor uncertainty modulates corticospinal excitability during skilled object manipulation

**DOI:** 10.1152/jn.00800.2018

**Published:** 2019-02-06

**Authors:** Marco Davare, Pranav J. Parikh, Marco Santello

**Affiliations:** ^1^Department of Movement Sciences and Leuven Brain Institute, KU Leuven, Leuven, Belgium; ^2^Sobell Department of Motor Neuroscience and Movement Disorders, Institute of Neurology, University College London, London United Kingdom; ^3^Department of Health and Human Performance, University of Houston, Houston, Texas; ^4^School of Biological and Health Systems Engineering, Arizona State University, Tempe, Arizona

**Keywords:** finger, forces, M1, transcranial magnetic stimulation

## Abstract

Sensorimotor memory built through previous hand-object interactions allows subjects to plan grasp forces. The memory-based mechanism is particularly effective when contact points on the object do not change across multiple manipulations, thus allowing subjects to generate the same forces in a feedforward fashion. However, allowing subjects to choose where to grasp an object causes trial-to-trial variability in fingertip positioning, suggesting a decreased ability to predict where the object will be grasped. In this scenario, subjects modulate forces on a trial-to-trial basis as a function of fingertip positioning. We suggested that this fingertip force-to-position modulation could be implemented by transforming feedback of digit placement into an accurate distribution of fingertip forces. Thus, decreasing certainty of fingertip position on an object would cause a shift from predominantly memory- to feedback-based force control mechanisms. To gain further insight into these sensorimotor transformation mechanisms, we asked subjects to grasp and lift an object with an asymmetrical center of mass while preventing it from tilting. To isolate the effect of digit placement uncertainty, we designed two experimental conditions that differed in terms of predictability of fingertip position but had similar average fingertip positioning and force distribution. We measured corticospinal excitability to probe possible changes in sensorimotor processing associated with digit placement uncertainty. We found a differential effect of sensorimotor uncertainty after but not before object contact. Our results suggest that sensorimotor integration is rapidly tuned after object contact based on different processing demands for memory versus feedback mechanisms underlying the control of manipulative forces.

**NEW & NOTEWORTHY** The relative contribution of predictive and feedback mechanisms for scaling digit forces to position during dexterous manipulation depends on the predictability of where the object will be grasped. We found that corticospinal excitability shortly after contact was sensitive to digit position predictability. This supports the proposition that distinct sensorimotor integration processes are engaged, depending on the role of feedback about digit placement versus sensorimotor memory in controlling manipulative forces.

## INTRODUCTION

Fine manipulative skills and dexterous tool use are unique attributes of the human species. Skilled object manipulation relies on the ability to coordinate fingertip forces flexibly, depending on where we intend to place our fingertips when grasping an object (Fu et al. 2010; Mojtahedi et al. 2015). This force-to-position coordination is crucial because it allows us the flexibility of grasping a given object in different ways and modulating forces accordingly to perform the same manipulation. For example, we can lift a glass of water straight without spilling its content regardless of where the glass is grasped, i.e., by placing the fingertips around the rim of the glass using a prismatic or using a cylindrical grasp, despite the fact that the two ways of grasping the glass require different finger force distributions. For tasks that allow choice of digit placement, we have proposed that successful force-to-position coordination depends on the interaction between predictive force control mechanisms and sensory feedback about digit placement (Mojtahedi et al. 2015). Predictive mechanisms in the form of sensorimotor memory can be used to predict fingertip contact points with the object and plan the appropriate fingertip force distribution (Gordon et al. 1993; Johansson and Cole 1992; Loh et al. 2010; Lukos et al. 2008; Westling and Johansson 1984). However, the extent to which the brain can rely on sensorimotor memory to use the same fingertip contact points and force patterns is significantly limited when subjects can grasp the object in many different ways. This is due to the fact that subjects exhibit trial-to-trial variability of fingertip positioning (Fu et al. 2010; Mojtahedi et al. 2015). This position variability, which results from motor planning and/or execution noise, is compensated for by trial-to-trial modulation of fingertip forces. One mechanism by which digit force-to-position modulation could be implemented is via online sensory feedback of digit placement, which would then be integrated on a trial-to-trial basis with predictive information to allow digit forces to be precisely modulated as a function of a given digit position. To date, however, it is still unclear how sensorimotor mechanisms rapidly integrate sensory feedback for online motor control.

**Fig. 1.**
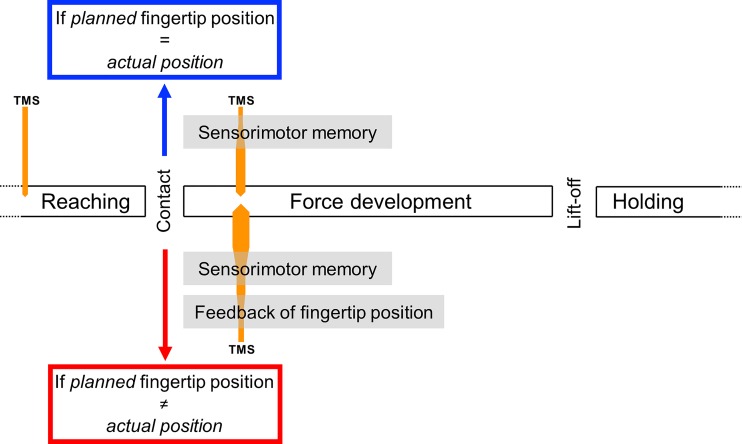
Theoretical framework for sensorimotor mechanisms underlying dexterous manipulation. Diagram shows 2 putative mechanisms responsible for controlling fingertip forces according to whether planned and actual fingertip positions at contact with an object match or do not match. When planned and actual fingertip positions match, as happens when contacts are constrained and invariant across trials (blue), sensorimotor memory built through previous manipulation is sufficient to retrieve fingertip forces. Conversely, when planned and actual fingertip position do not match (red), integration of feedback about digit positions is crucial to modulate forces up to object lift onset. In both cases, object manipulation is accurately performed by attaining the desired fingertip force distribution at object lift onset, i.e., the compensatory torque required to prevent the object from tilting. The transcranial magnetic stimulation (TMS) time points for probing corticospinal excitability (CSE) during midreach and after object contact are also shown, with the thickness of the probe representing hypothesized CSE changes (orange).

The present experiments addressed this gap by using a task in which we controlled the relative contribution of predictive digit force control mechanisms and online sensory feedback about digit placement. Subjects were asked to use the thumb and index fingertip to lift an object with an asymmetrical center of mass without tilting it. We varied the uncertainty in predicting digit placement by either visually cueing grasp contact points on the object (high predictability) or allowing subjects to freely choose their grasp contact points (low predictability) while keeping the overall fingertip positioning and force distribution similar across two experimental conditions. Our rationale was that when grasp locations were highly predictable, fingertip forces and grasp locations could be planned well before contact time and that the forces would rely more on sensorimotor memory than feedback mechanisms (Fig. 1) (Johansson and Cole 1992; Johansson and Flanagan 2009; Westling and Johansson 1984). Conversely, when grasp locations were not predictable, fingertip forces would critically rely upon rapid integration of sensory feedback about digit placement just after object contact (Fu et al. 2010; Mojtahedi et al. 2015). This comparison between high and low predictability of grasp locations, especially when motor output is statistically indistinguishable, would thus unveil an important feature of sensorimotor integration mechanisms; sensorimotor integration processes responsible for grasp forces can be dynamically adjusted, depending on the relative role of sensory inputs and memory. We measured the amplitude of motor-evoked potentials elicited by single-pulse transcranial magnetic stimulation to examine differences in sensorimotor integration processes under low- and high-predictability conditions with identical motor output. We hypothesized that, despite the manipulation task being characterized by similar motor output and performance, corticospinal excitability would be significantly modulated as a function of fingertip position predictability and hence, the extent to which fingertip force control would rely predominantly on memory versus digit position feedback. Our theoretical framework (Fig. 1) proposes a greater reliance on sensory feedback of digit placement in the low-predictability condition. At the cortical level, this would lead to a greater processing load of sensory inputs from S1 than in the high-predictability condition. Thus, the hypothesized difference in corticospinal excitability (CSE) between the two experimental conditions would reflect a greater sensitivity (excitability) of sensory and motor neurons in terms of processing incoming proprioceptive and tactile inputs and processing S1 inputs to scale digit forces accordingly, respectively. This difference in sensorimotor integration processes is illustrated in Fig. 1, where changes in the thickness of the TMS probe represent hypothesized changes in CSE, depending on the processing demands in the low- versus high-predictability conditions, and after-object contact versus mid-reach (Lemon 1981; Lemon et al. 1995).

## METHODS

### Participants

Twelve naïve, right-handed (Oldfield 1971) volunteers aged between 20 and 28 yr (means ± SD: 23 ± 2 yr; 5 females) with normal or corrected-to-normal vision, no history of neurological disease, musculoskeletal disorders, or upper limb injury provided consent to participate in the study. Subjects were screened for potential risk of adverse reactions to transcranial magnetic stimulation according to the published guidelines (Rossi et al. 2009) using the TMS Adult Safety Screen (Keel et al. 2001). The participants were recruited from the pool of undergraduate and graduate students at Arizona State University. They received $10 as compensation for their participation. The experimental protocols were approved by the Office of Research Integrity and Assurance at Arizona State University.

### Apparatus

#### Grip device.

**Fig. 2.**
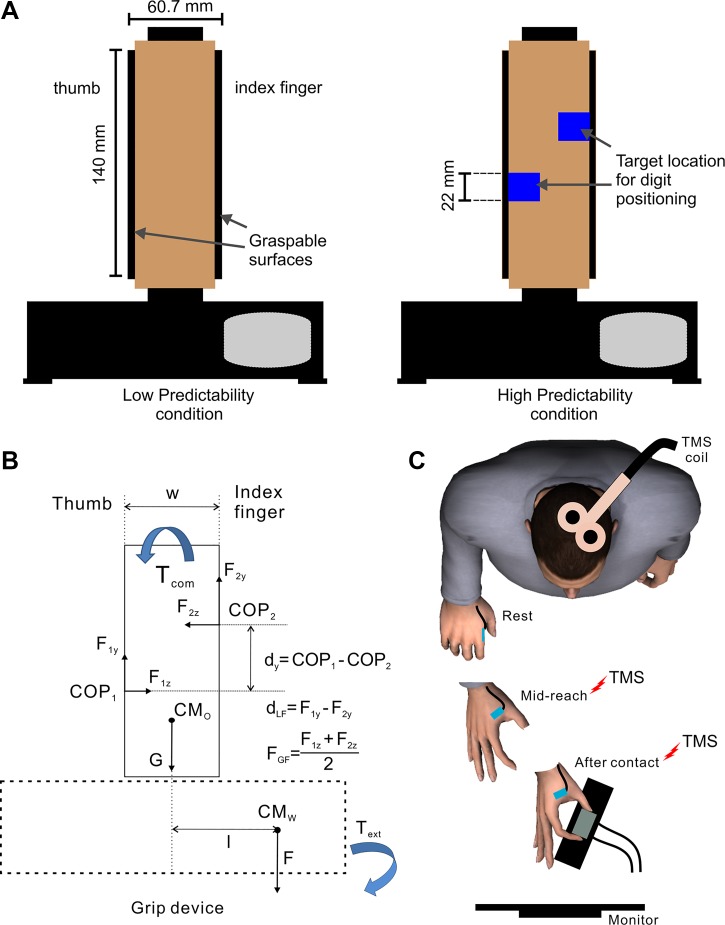
Experimental setup. *A*: grip device used for the 2 experimental conditions. In the high-predictability condition, blue markers indicated participants where to place the thumb and index fingertips. *B*: object body diagram shows forces acting on the object, gravity (G, F) acting on the object center of mass (CM_O_) and added mass (CM_w_), and forces exerted by the thumb (F_1_) and index fingertip (F_2_) in the normal (z) and tangential (y) directions through their centers of pressure (COP_1_ and COP_2_, respectively). The vertical distance between COP_1_ and COP_2_ is defined as d_y_, the difference between F_1y_ and F_2y_ is defined as d_LF_, and the average of F_1z_ and F_2z_ is defined as grip force (F_GF_). Subjects were required to exert a compensatory moment (T_com_) to counter the external moment (T_ext_) caused by the 400-g mass (CM_w_) inserted in the rightmost compartment acting at a distance (I) from the center of the object. *C*: single-pulse transcranial magnetic stimulation (TMS) was applied over the left M1 at 2 time points: midreach and shortly after contact.

This device has been described in detail elsewhere (Fu et al. 2010; Lukos et al. 2007; Zhang et al. 2010). Briefly, force and torques exerted by thumb and index finger were measured by two six-dimensional force/torque sensors (Nano-25; ATI Industrial Automation, Garner, NC) mounted on a custom-designed inverted T-shaped frame (Fig. 2*A*). The grip surfaces consisted of two plates covered with sandpaper, each mounted vertically on the thumb and index finger transducers. As described in our previous studies, the design of our grip device allows measurement of grip and load force (normal and tangential to the graspable surface) as well as the center of pressure of each digit. The base of the grip device consisted of three compartments (left, center, and right). For the present study, a 400-g mass was inserted in the rightmost compartment to shift the mass distribution of the grip device to the right of its vertical midline to generate an external torque of 255 Nmm on the object (Fig. 2*B*). The mass location was blocked by a lid to prevent visual identification of the object’s mass distribution. The total mass of the grip device, including the external mass, was 790 g. Each end of the base of the object was placed on a custom-made switch (object switches) to estimate object lift onset (i.e., onset of manipulation). The subject’s hand rested on a hand switch taped to the table to ensure repeatable start position and arm posture across trials and detect reach onset.

#### Experimental task.

Subjects were instructed to reach and grasp the grip device placed on a table 30 cm in front of them, using the thumb and index fingertips at a self-selected speed, lift it vertically to a height of ∼10 cm by trying to minimize the object from tilting, hold it for ∼1 s, and replace it on the table (Fig. 2*C*). We varied the amount of predictive information about digit placement by either grasp cueing contact points on the object (high predictability) or allowing subjects to freely choose grasp contact locations (low predictability). For the low-predictability condition, subjects were instructed that they could grip the object anywhere on its vertical graspable surfaces. For the high-predictability condition, subjects were instructed to grip the object at fixed locations indicated by markers placed on each side of the object facing the subject (Fig. 2*A*). We used black electrical tapes as the digit placement markers for the high-predictability condition. Tape color and size were the same across all sessions. A computer monitor placed behind the grip device was used to present visual cues to the subject. The first “ready” cue signaled the beginning of a trial and was followed by a “condition” cue to inform the subject about whether the upcoming condition was to be performed with a high or low predictability condition. Finally, a “go” cue was given 1 s after the “task” cue to instruct subjects to reach and perform the grasp-and-lift task. To ensure that subjects were tested after having learned the object dynamics, they performed 15 practice trials of the low-predictability condition. Subsequently, half of the subjects performed the grip and lift task first in the low-predictability condition followed by the high-predictability condition, and the other half performed the experiment in the opposite order. Subjects performed 42 trials each for the low-predictability condition and the high-predictability condition. Note that the locations of the digit placement markers for the high-predictability condition were estimated based on the digit center of pressure spontaneously adopted by each subject during the practice trials (low-predictability condition) if they were tested first on the high-predictability condition or in the low-predictability trials if they were first tested on the low-predictability condition. The rationale for asking subjects in the high-predictability condition to grasp at the same contact points they chose for the low-predictability condition was to ensure that subjects exerted the same normal and tangential digit forces in the two conditions.

### Transcranial Magnetic Stimulation

Resting motor threshold (rMT) was estimated using single monophasic magnetic pulses delivered, with a 50-mm figure-of-eight coil connected to a Magstim model 200 stimulator (Magstim, Whitland, UK) (Parikh et al. 2014). With the TMS coil held tangential to the scalp and at 45° from the midsagittal line, suprathreshold TMS pulses were delivered to the left M1 region containing the cortical representation of the right first dorsal interosseus muscle (FDI). The TMS coil position was maneuvered to optimize the motor-evoked potential (MEP) amplitude in all recorded muscles (Davare et al. 2009; Parikh et al. 2014; Parikh and Santello 2017). The rMT was determined as the TMS intensity that induced 50 μV peak-to-peak MEPs in five of 10 trials in relaxed FDI muscle (Rossi et al. 2009; Parikh et al. 2014). The average rMT was 41 ± 2% (means ± SE) of the maximal stimulator output. During all experimental procedures, the TMS coil was stabilized using a coil holder mounted on the TMS chair (Rogue Research).

**Fig. 3.**
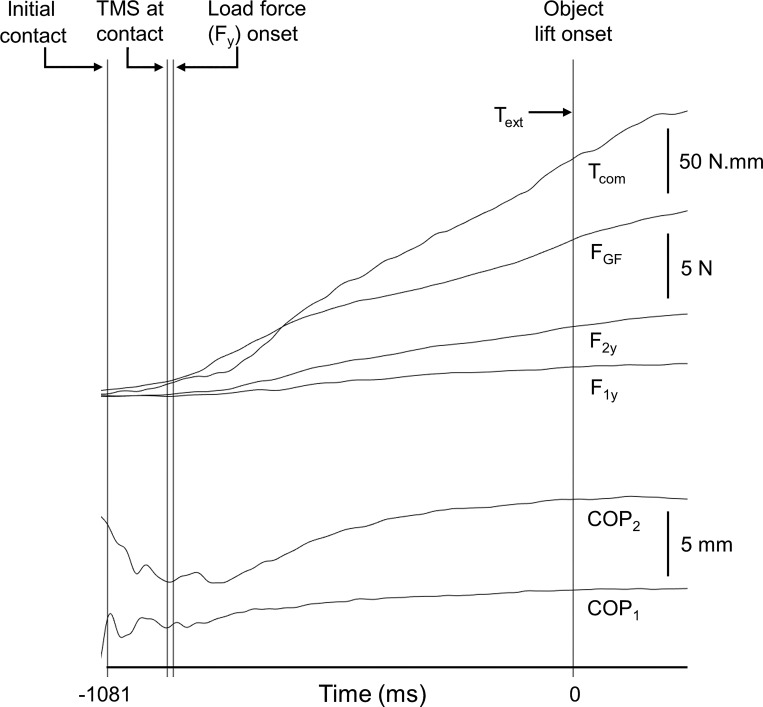
Timing of transcranial magnetic stimulation (TMS) relative to behavioral events. The events of contact, onset of load force, and object lift onset from 1 trial are shown together with the time course of grip force (F_GF_), thumb (F_1y_), and index finger (F_2y_) tangential forces, compensatory torque (T_com_) required to counter the external torque (T_ext_), and thumb (COP_1_) and index finger (COP_2_) centers of pressure. To facilitate comparison, T_com_ is plotted with a positive sign. Delivery of single-pulse TMS was timed relative to initial contact, which was defined as the instant at which F_GF_ was >1 N. The TMS delivery time relative to initial contact averaged across all subjects was 118 ms. The onset of LF averaged across all subjects occurred 12 ms after TMS. For graphical purposes, the scale of the T_com_ calibration bar was reduced 10-fold relative to the force calibration bar.

We delivered single-pulse TMS at 80% rMT in 32 out of the 42 trials in a block at two time points in a pseudorandom order *1*) during the reach (16 trials) and *2*) just after contact with the object (16 trials; see Fig. 2*C*). TMS during the reach was delivered when the time from reach onset was 50% of the mean reach duration averaged across the last 10 practice trials. For reliable and consistent detection of the object contact event, TMS at contact was delivered 20 ms after the grip force exerted on the object reached 1 N (Fig. 3). We used an 80% rMT TMS intensity to avoid MEP size saturation in muscles that would become active just before and at object contact (Lemon et al. 1995). It is unlikely that the TMS pulse over the M1 “hotspot” for the FDI muscle also stimulated the neighboring hand primary somatosensory cortex (S1), which is known to be located ∼2.5 cm posterior to the M1 hand area (Bolognini et al. 2011; Lockwood et al. 2013). This is because we used a small TMS coil that produces focal electric fields (Deng et al. 2013), delivered TMS pulses at a submotor threshold intensity that minimizes current spread to surrounding structures (Siebner et al. 2009), and oriented the TMS coil such that the induced current in the brain was postero-anteriorly directed.

### Data Acquisition and Analysis

We recorded electromyographic (EMG) activity of right-hand muscles using differential surface electrodes (Delsys Bagnoli EMG System, Boston, MA) from first dorsal interosseus (FDI), abductor pollicis brevis (APB), abductor digiti minimi (ADM), and flexor carpi radialis (FCR). The reference electrode was placed on the dorsum of the right wrist. Prior to electrode placement, the skin was prepared using isopropyl alcohol pads. The experimenter verified that the electrodes remained in place throughout the experiment.

The peak-to-peak amplitude of motor-evoked potentials (MEP) was measured from all the above muscles to quantify CSE during preparation for and execution of the two grip-lift conditions. Importantly, to avoid background EMG activity to influence MEP amplitude, MEP size was normalized relative to the background EMG activity (root-mean-square) occurring 100 ms before the TMS pulse (Lemon et al. 1995). Less than 5% of MEP data that fell outside 3 SD of the mean were identified as outliers and removed from further analysis.

EMG data were acquired by a 16-bit A/D Micro1401 board using Spike2 software (sampling frequency: 2 kHz; CED). Force and torque data were acquired by 12-bit A/D converter boards using LabVIEW software (sampling frequency: 1 kHz, PCI-6225; National Instruments, Austin, TX). We analyzed the following variables: *1*) digit forces [digit load force (F_y_) is the vertical force component parallel to the grip surface produced by each digit to lift the object, and digit normal force (F_z_) is the force component perpendicular to the grip surface]; and *2*) digit center of pressure for each digit, which was defined as the vertical coordinate of the point of resultant force application produced by the digit on the grip surface (COP) (Fig. 2*B*). As was done in our previous work (Fu et al. 2010; Zhang et al. 2010), digit force and center of pressure data were used to compute the average of grip forces exerted by the two digits (F_GF_), the difference between load forces exerted on the thumb and finger side of the grip device (d_LF_), and the vertical distance between the center of pressure on the thumb and finger side of the grip device (d_y_). These three variables were used to compute the compensatory torque (T_com_), defined as the torque used to minimize object roll exerted at object lift onset by modulating F_GF_, d_LF_, and d_y_ to balance the external torque caused by the added mass (T_ext_) (Fig. 2*B*).

As was done in our previous work (Fu et al. 2010; Zhang et al. 2010), we computed T_com_ exerted by the subject on the object as follows:

1 $$T_{com}=\left(\frac{\text{w}}{\text{2}}\times {\text{d}}_{\text{LF}}\right)\text{+}({\text{d}}_{\text{Y}}\times {\text{F}}_{\text{GF}}),$$

where d_LF_ denotes the difference between thumb and index finger load force, d_Y_ denotes the vertical distance between thumb and index finger center of pressure, F_GF_ denotes the grip forces, w denotes the grip width, and $\frac{\text{w}}{\text{2}}$ denotes half-width of the object THAT corresponds to the moment arm for the thumb and index finger load force.

All of these performance variables were computed at object lift onset. Object lift was the time when the first of the two object switches was released and remained open for 50 ms. Reach onset time was the time when the hand switch was released and remained open for 50 ms. Reach duration was the time from reach onset time to object initial contact. Initial digit contact was defined as the time at which grip force produced by both digits crossed and remained above a threshold (mean + 2 SD of the signal baseline) for 50 ms. The load force onset was defined as the time at which load force produced by both digits crossed and remained above a threshold (mean + 2 SD of the signal baseline) for 50 ms. The load force onset occurred on average 130 ± 17 ms following the digit initial contact. TMS at contact was delivered on average 118 ± 15 ms following the digit initial contact (means ± SE across all trials where TMS was delivered at contact). TMS timing relative to behavioral events are shown in Fig. 3.

### Grip Force Rate Analysis

We performed continuous wavelet transform (CWT) analysis on grip force rate during the loading phase (contact to object lift onset) to quantify the relative intensity of corrective force responses during trials of the high- and low-predictability conditions (Mojtahedi et al. 2015). Briefly, we computed CWT as the integral of grip force rate over the duration of loading phase multiplied by the scaled and shifted versions of the Mexican hat wavelet function. The goal of this analysis was to identify bell-shaped profiles in the grip force rate. Bell-shaped profiles of motor variables, e.g., wrist position during rapid reaching movements or grip force used to lift objects of known mass, are thought to be driven by feedforward control. Conversely, profiles that are characterized by corrective, feedback-driven responses, e.g., sudden target changes during reaching or object mass across trials, would exhibit irregular trajectories (Ghez et al. 1995; Ghez and Gordon 1987; Gordon et al. 1993, 1995; Jeannerod 1984; Jenmalm and Johansson 1997; Johansson and Westling 1988). The Mexican hat wavelet function is bell shaped. Therefore, CWT quantified the extent to which force profiles resembled the bell-shaped function across time and frequencies. As was done in Mojtahedi et al. (2015), we computed the slow and fast bell-shaped components using the scale and translation parameters derived from CWT. R_avg_ was defined as the ratio of slow bell-shaped component to the sum of slow and fast bell-shaped components. The slow bell-shaped component is characterized by lower pseudofrequencies (2.08 to 3.125 Hz), whereas the fast bell-shaped component is characterized by higher pseudofrequencies (9.09 to 14.28 Hz). In this CWT framework, the slow bell-shaped component captures the low-frequency feedforward component of force control, whereas the fast bell-shaped component captures high-frequency, feedback-driven corrective force responses. Thus, larger and smaller R_avg_ values denote a greater similarity or dissimilarity, respectively, between the grip force rate profile and a bell-shaped profile, thereby denoting a more feedforward- or feedback-driven grip force control.

### Statistical Analyses

We performed repeated-measures ANOVA on normalized MEP and background EMG with predictability condition (low, high), TMS delivery time points (midreach, contact), and muscles (FDI, APB, ADM, FCR) as within-subject factors. We applied Huynh-Feldt corrections when sphericity assumption was violated. Post hoc pairwise comparisons were performed with appropriate corrections. As stated earlier, we hypothesized that despite the manipulation task being characterized by similar motor output and performance, MEP would be significantly modulated as a function of fingertip position predictability. Therefore, we planned paired *t*-tests with Bonferroni corrections to compare for MEP between low- and high-predictability conditions at object contact and during midreach. Comparisons of d_LF_, d_y_, T_com_, and R_avg_ between low- and high-predictability conditions were performed using paired *t*-tests. We also performed linear regression analysis to quantify the correlation between d_y_ and d_LF_ over all trials within each predictability condition. Prior to computing Pearson’s correlation coefficients (*r*), we normalized d_y_ and d_LF_ for each subject by removing the mean of all trials from the value of each trial and dividing the result by the SD of the mean. Table 1 reports mean and standard deviation for all variables. The significance level was set at *P* < 0.05 (SPSS version 21; SPSS, Chicago, IL).

**Table 1.** All variables

Variable	Low Predictability	High Predictability
T_com_, Nmm	−201.9 ± 24.8	−205.5 ± 27.8
d_y_ Magnitude, mm	−7.09 ± 7.97	−8.78 ± 8.55
d_LF_ magnitude, N	−4.02 ± 2.26	−3.53 ± 2.76
F_GF_ magnitude, N	11.44 ± 2.64	11.9 ± 2.78
d_y_ Variability, mm	2.92 ± 0.88	2.16 ± 0.4
R_avg_	77.69 ± 3.61	79.38 ± 3.03
Thumb COP variability, mm	4.62 ± 2.48	1.84 ± 0.24
Index finger COP variability, mm	5.4 ± 2.61	1.96 ± 0.45
Normalized MEP FDI at contact	75.71 ± 66.5	64.45 ± 48.9
Normalized MEP FDI during mid reach	5.09 ± 4.55	5.11 ± 4.16
Normalized MEP APB at contact	7.69 ± 6.38	7.66 ± 5.48
Normalized MEP APB during mid reach	8.36 ± 5.64	8.62 ± 5.8
Normalized MEP ADM at contact	12.5 ± 8.3	12.2 ± 7.03
Normalized MEP ADM during mid reach	7.17 ± 4.67	5.43 ± 4.28
Normalized MEP FCR at contact	5.31 ± 2.15	5.15 ± 2.11
Normalized MEP FCR during mid reach	4.46 ± 2.47	3.46 ± 1.58

Values are means ± SE. APB, abductor pollicis brevis; ADM, abductor digiti minimi; COP, centers of pressure; d_LF_, fingertip load force distribution; d_y_, digit position; FCR, flexor carpi radialis; FDI, first dorsal interosseous; F_GF_, normal force; MEP, motor-evoked potential; R_avg_, bell-shaped components of grip force; T_com_, compensatory torque.

## RESULTS

Based on previous observations (Fu et al. 2010), we expected greater trial-to-trial variability in digit placement in the low- than in the high-predictability condition. This, in turn, should have led to modulation of digit forces to fingertip position on a trial-to-trial basis in only the low-predictability condition. Despite this between-condition difference in digit position variability, we expected subjects to attain the same compensatory torque (T_com_) in both low- and high-predictability conditions. Our results confirmed this expectation; subjects were able to lift the object (Fig. 2*A*) while preventing it from tilting equally well in both the low- and high-predictability task conditions, as indicated by similar T_com_ at object lift onset (mean difference = 3.6; paired *t*-test; *t*_11_ = 1.29, *P* = 0.22).

### Validation of Experimental Protocol

**Fig. 4.**
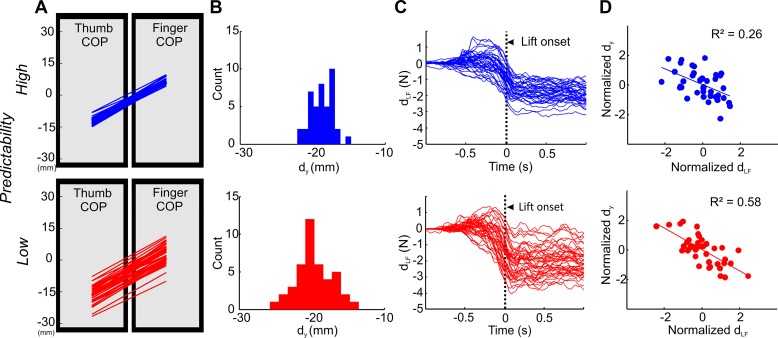
Representative digit position and force data. Behavioral data from 1 representative subject performing a grasp and lift of the device under high- and low-predictability conditions (blue and red lines and symbols, respectively). *A*: thumb and index finger center of pressure (COP) on the graspable surfaces. Each line denotes a trial. *B*: distribution of vertical distances between thumb and index finger COP (d_y_). *C*: difference between thumb and index finger load force (d_LF_); “0” on the time axis denotes contact, and the vertical dotted line denotes object lift onset. Each line denotes a trial. *D*: the relation between normalized d_y_ and normalized d_LF_ is shown with a linear fit and the coefficient of determination.

The interpretation of the effects of TMS on CSE required our task to elicit comparable average digit position and force distributions in both task conditions despite a greater variability in digit position in the low-predictability condition. Our results confirmed these expectations and validated our experimental protocol. Specifically, digit position and force distributions at object lift onset were similar in both conditions; digit position (d_y_; Fig. 4, *A* and *B*), fingertip load force distribution (d_LF_; Fig. 4*C*), normal force (F_GF_), and T_com_ in the low- and high-predictability conditions were statistically indistinguishable (*t*-tests; all *P* values ≥0.15). Importantly, the low-predictability condition exhibited greater trial-to-trial digit position variability than the high-predictability condition (mean difference = 0.76; *t*_11_ = 2.8, *P* = 0.018), a finding consistent with our earlier work (Fu et al. 2010). Furthermore, the background muscle activity was not significantly different across low- and high-predictability conditions (*P* = 0.46). Altogether, these results show that subjects’ motor behaviors in both the low- and high-predictability condition were similar despite the former condition being characterized by greater digit position variability.

### Digit Force-to-Position Modulation and Corrective Force Responses Were Predominantly Present in Low- Relative to High-Predictability Condition

**Fig. 5.**
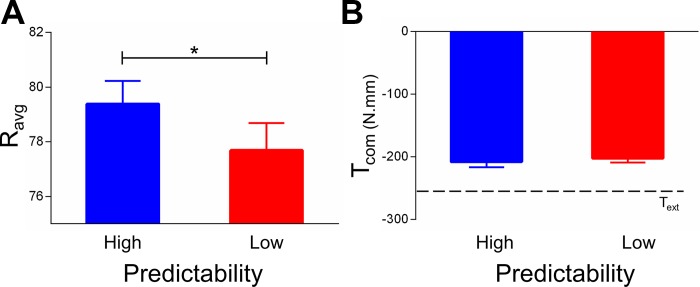
Analysis of grip force and compensatory torque. *A*: ratio of slow vs. fast bell-shaped components of grip force (R_avg_) obtained from continuous wavelet transform analysis performed from contact to object lift onset for both experimental conditions. Smaller values denote greater extent of corrective force responses. *B*: compensatory torque. Compensatory torque (T_com_) exerted on the object is plotted relative to the target torque (T_ext_) for both experimental conditions. Data are averages (± SE) of all subjects. *Statistically significant difference at *P* < 0.05.

Despite subjects attaining the same T_com_ in both grasp conditions, as expected from our previous work (Fu et al. 2010), the trial-to-trial covariation between d_y_ and d_LF_ was significantly stronger in the low- than in the high-predictability condition (mean difference = 0.15; *t*_11_ = 3.45, *P* = 0.005; Fig. 3*D*). Furthermore, we characterized the role of feedback and predictive mechanisms in the control of digit forces by performing continuous wavelet transform (CWT) analysis of grip force profiles (Mojtahedi et al. 2015). Consistent with our recent observations (Mojtahedi et al. 2015), CWT of grip force rate profiles measured between contact and object liftoff revealed a greater occurrence of feedback-driven corrective force responses for the low- than for the high-predictability condition (mean difference = −1.69; significantly smaller R_avg_; *t*_11_ = −2.26, *P* = 0.04; Fig. 5*A*). This result suggests that sensory feedback of digit placement played a greater role in the low-predictability condition. We note that, regardless of differences in how digit forces developed from contact to object lift onset (Fig. 5*A*), subjects attainted the same T_com_ at object lift onset (Fig. 5*B*).

### Effects of Single-Pulse TMS on Corticospinal Excitability

**Fig. 6.**
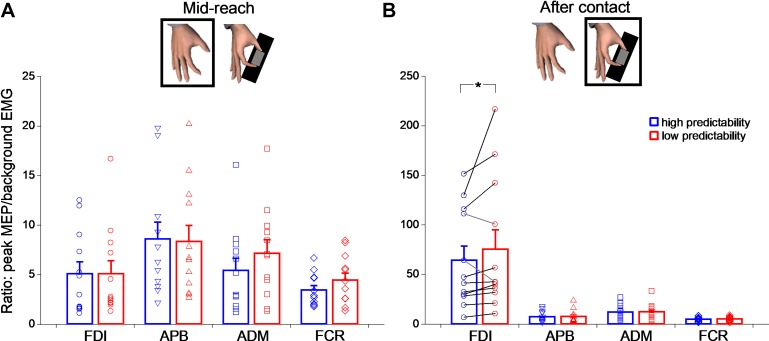
Changes in corticospinal excitability (CSE) as a function of task epoch, muscle, and experimental condition. *A*: CSE at the “midreach” time point, measured as the motor-evoked potential (MEP) amplitude normalized relative to the 100-ms root-mean-squared electromyographic (EMG) background for first dorsal interosseous (FDI), abductor pollicis brevis (APB), abductor digiti minimi (ADM), and flexor carpi radialis (FCR) muscles and for the low- (red) and high-predictability (blue) conditions. Bar plots show all subjects’ averages and SE. Open symbols represent each individual subject average. *B*: same as *A*, but for TMS applied after contact. Note a 10-fold MEP amplitude increase specifically for the FDI just after object contact, for which we also found a significant effect of digit position predictability (*P = 0.008).

The above findings point to *1*) identical motor output in two grasp conditions despite greater digit position variability in the low-predictability condition and *2*) different contributions of feedback versus predictive digit force control mechanisms. Both observations are critically important, as they allowed us to test the hypothesis that predictability of digit position would modulate motor-evoked potential (MEP) using single-pulse transcranial magnetic stimulation (TMS). Given these observations, we would interpret between-condition differences in MEP stemming from differences in sensorimotor integration mechanisms underlying grasp force control. We applied TMS at two different time points during the task: before and just after object contact. Consistent with previous work (Lemon et al. 1995), we found that MEP amplitude at object contact was greater than during midreach (ANOVA_RM_; main effect of time: *F*_1, 11_ = 16.49, *P* = 0.002; ƞ_p_^2^ = 0.6, observed power = 0.96; Fig. 6, *A* vs. *B*). Moreover, a main effect of muscle (*F*_1.054, 11.59_ = 15.37, *P* = 0.002, ƞ_p_^2^ = 0.58, observed power = 0.95) and muscle × time interaction (*F*_1.029, 11.32_ = 15.16, *P* = 0.002, ƞ_p_^2^ = 0.58, observed power = 0.95) showed that this increase in CSE from the midreach to the object contact time points was different across muscles. Post hoc analyses revealed that MEP size increased further in the FDI compared with the APB, ADM, and FCR (mean difference 62.4, 57.7 and 64.8, respectively; paired *t*-test with Bonferroni correction: all *t*_11_ > 5.178, all *P* < 0.001; Fig. 6*B* and Table 1). This suggests that CSE changes between midreach and object contact are muscle specific and target mainly the FDI representation.

Next, we performed planned comparisons to investigate the effect of fingertip position predictability for each muscle. Interestingly, we found that FDI MEP amplitude at object contact was significantly larger when predictability about grasp locations was low compared with when predictability was high (mean difference = 11.26; paired *t*-test with Bonferroni correction: *t*_11_ = 2.79, *P* = 0.008; Fig. 6*B*). The effect of fingertip position predictability on MEP amplitude at object contact was not significant for all the other muscles (paired *t*-test with Bonferroni correction: all *t*_11_ < 0.0068, all *P* > 0.99; Fig. 6*B*) Finally, MEP amplitude probed during midreach did not reveal any effect of grasp predictability in any muscle (all *P* values >0.2; Fig. 6*A*).

To understand why predictability of grasp locations affected CSE only in the FDI muscle, we examined whether the two digits contributed equally to the trial-to-trial variability in net digit placement variability. As expected (Fu et al. 2010) and reported above, trial-to-trial variability of the vertical distance between thumb and index fingertip (d_y_) was greater for the low- than the high-predictability condition (Fig. 4, *A* and *B*). Interestingly, however, this difference was driven mostly by the greater index fingertip contact point variability relative to that of the thumb for the condition with low predictability (mean difference = 0.78; *t*_11_ = 2.924, *P* = 0.014). In contrast, for the condition with high predictability about digit placement, there was no difference between thumb and index finger contact point variability (mean difference = 0.12; *t*_11_ = 1.147, *P* = 0.3). Thus, the effect of predictability of grasp location on CSE might reflect a specific role of the index fingertip relative to the thumb in our task, i.e., the thumb could have been used consistently as a “pivot” relative to a more variable positioning of the index fingertip.

## DISCUSSION

The present study used a unique behavioral paradigm to vary the amount of predictive information about digit placement on an object, thus allowing us to investigate the extent to which corticospinal excitability might reveal differences in sensorimotor integration processes. Based on the theoretical framework we proposed in our previous work (Fu et al. 2010; Mojtahedi and Santello 2014) (Fig. 1), in the case of low predictability of grasp locations, the sensorimotor system must, following object contact, *1*) monitor potential mismatch between expected and actual finger position and *2*) integrate sensory information regarding digit position rapidly for accurate modulation of digit forces.

The across-trial variability in the low-predictability condition indicates that digit forces must be scaled according to the current rather than previous digit placement, hence, underscoring a significant role of feedback of digit placement for digit force modulation. In contrast, when prior information about grasp locations is available (i.e., contact points are highly predictable due to the fact that they do not change across trials), force coordination can be controlled using feedforward mechanisms and depends relatively less on feedback processing of digit placement (Gordon et al. 1993; Johansson and Cole 1992; Johansson and Flanagan 2009; Westling and Johansson 1984). This proposition is supported by our present and previous observations based on continuous wavelet transforms of grip force rate profiles showing that high-frequency force corrections occur to a significantly greater extent in conditions with less predictable grasp locations (Mojtahedi et al. 2015).

### Sensorimotor Transformations for Grasp Force Control

Here, we probed corticospinal excitability so as to quantify the differences in sensorimotor integration mechanisms under two conditions with similar motor output (i.e., no difference in the magnitude of load force distribution at the time of TMS or at object lift onset). We found that decreasing predictability in fingertip positioning enhanced CSE at object contact and only for a hand muscle primarily involved in the control of our task. This suggests that distinct sensorimotor integration processes were engaged based on the demands for feedback of digit placement required for achieving the action goal. Our effect of predictability of grasp locations on CSE is consistent with a differential weighting between predictive and feedback mechanisms for digit force control during dexterous manipulation. This finding underscores the functional significance of the contact event for triggering the processing of sensory inputs caused by physical hand-object interaction. Although similar context-dependent CSE modulation was not observed during the reach, it is possible that visual and somatosensory feedback of the hand just before contact might play a role in anticipating final digit placement, hence digit force distribution. This visual information might have influenced corticospinal excitability before object contact in anticipation to perform adjustments in digit force distribution for the low-predictability condition (Loh et al. 2010). However, our study was not designed to assess context-specific modulation of CSE just before contact. Together, findings obtained from TMS delivered during the reach versus contact suggest that the CSE modulation at object contact is related to uncertainty in final fingertip positioning and differential sensorimotor integration processing primarily involving M1 and S1. Given that the exact moment of contact may not be predictable with a high degree of accuracy, we should note that CSE modulation at object contact might have started around the predicted time of contact, i.e., within a time window shortly before and after contact, rather than starting exactly at contact. For the time period just before object contact, it remains unknown whether sensory information about digit trajectory can be used to anticipate the final digit position and digit force distribution. Even if it did, digit position variability (Fig. 4, *A* and *B*) and grip force rate analysis (Fig. 4*A*) indicate that such anticipation is noisy and requires corrections to attain the required T_com_ at object lift onset (Fig. 5*B*). Based on the finding that more corrective load force responses occur in the low- than in the high-predictability condition between object contact and lift onset, one may argue that CSE modulation might have been caused by differences in motor execution from contact to object lift onset. However, we rule out this explanation, as CSE was probed by TMS ∼10 ms before onset of load force (see methods and Fig. 3).

It has been proposed that tactile feedback signals following contact are used to monitor task progression and compared with signals expected at distinct task epochs such as initial object contact or object liftoff (Johansson and Flanagan 2009). This sensorimotor control point hypothesis was based on tasks that allow high predictability of grasp locations, thus allowing predictive scaling of digit forces. In contrast, our low-predictability task, resulting in greater trial-to-trial variability in contact points and requiring appropriate digit force modulation irrespective of the object dynamics (i.e., whether the object’s mass is symmetrically or asymmetrically distributed), demonstrates distinct neural processing at object contact. This strongly supports our theoretical framework that dexterous manipulation may also require online monitoring of digit positioning at and following object contact (Fu et al. 2010, 2011; Mojtahedi et al. 2015; Santello 2018) and its generalization to a wider range of hand-object interactions beyond the task studied here.

In our study, the delay between the initial object contact and TMS was ∼120 ms, which is consistent with the delay required for both somatosensory as well as visual signals to influence M1 neurons. Recently, it has been shown that M1 is able to utilize afferent feedback in generating fast corrective movements of ∼50 ms following a perturbation (Pruszynski et al. 2011). On the other hand, the visual feedback has been found to influence the corticospinal system in as little as 50 ms after object presentation for anticipatory grasp force control (Loh et al. 2010). In the low-predictability condition, a combination of visual and somatosensory feedback at contact might have been crucial to trigger a series of events to compensate for trial-to-trial variability in digit positioning, specifically *1*) sensing actual digit position and possibly comparing this feedback with anticipated digit position, *2*) enabling digit force modulation as a function of sensed digit placement, and *3*) achieving the target moment required for minimizing object tilt. Thus, sensory feedback under the low- versus high-predictability condition might have induced a greater response in M1 in the form of greater excitability of corticospinal tract (Lemon 1981). This larger neuronal activity resulted in larger TMS evoked responses in the low- than in the high-predictability condition following contact (Lemon 1981; Lemon et al. 1995). Consistent with this argument, we observed larger response to TMS in the index finger muscle (FDI), but not in the thumb muscle (APB), because the index finger was the key contributor to the greater trial-to-trial variability in digit positioning in the low-predictability condition. It is also noteworthy that the CSE modulation we observed is more likely to originate from M1 rather than spinal circuitry, considering the delay between object contact and TMS (∼120 ms). Although this study focused on the grasp/context-specific role of M1 as the final stage of sensorimotor processing, a recent study identified additional brain areas that are uniquely involved with digit force-to-position modulation, e.g., cerebellar lobule I–IV, Broca area 44, and PMv (Marneweck et al. 2018).

In conclusion, we demonstrate that the sensorimotor integration processing depends on the relative role of sensory- and memory-based mechanisms for controlling manipulative forces and can be tracked by measuring corticospinal excitability at object contact. It is noteworthy that this sensory modulation occurred in conditions with strictly comparable motor outputs, thus ruling out any motor confound. This suggests that corticospinal excitability may not always be directly correlated with a motor output, as it can also probe different sensorimotor processes leading to identical motor output.

## GRANTS

This research was partially supported by a Collaborative Research Grant BCS-1455866 (M. Santello) from the National Science Foundation, a BBSRC (BB/J014184/1, UK) David Phillips fellowship (M. Davare), and a Fonds Wetenschappelijk Onderzoek grant (M. Davare; FWO Odysseus, G/0C51/13N, Belgium).

## DISCLOSURES

No conflicts of interest, financial or otherwise, are declared by the authors.

## AUTHOR CONTRIBUTIONS

M.D., P.J.P., and M.S. conceived and designed research; M.D., P.J.P., and M.S. analyzed data; M.D., P.J.P., and M.S. interpreted results of experiments; M.D., P.J.P., and M.S. prepared figures; M.D., P.J.P., and M.S. drafted manuscript; M.D., P.J.P., and M.S. edited and revised manuscript; M.D., P.J.P., and M.S. approved final version of manuscript; P.J.P. performed experiments.
